# Antimicrobial activity of the essential oil of *Tetradenia riparia* (Hochst.) Codd. (Lamiaceae) against cariogenic bacteria

**DOI:** 10.1590/S1517-838246246220140649

**Published:** 2015-06-01

**Authors:** Nathalya Isabel de Melo, Carlos Eduardo de Carvalho, Letícia Fracarolli, Wilson Roberto Cunha, Rodrigo Cassio Sola Veneziani, Carlos Henrique Gomes Martins, Antônio Eduardo Miller Crotti

**Affiliations:** 1 Universidade de Franca, Núcleo de Pesquisas em Ciências Exatas e Tecnológicas, Universidade de Franca, Franca, SP, Brasil, Núcleo de Pesquisas em Ciências Exatas e Tecnológicas, Universidade de Franca, Franca, SP, Brazil.; 2 Universidade de São Paulo, Faculdade de Filosofia, Ciências e Letras de Ribeirão Preto, Universidade de São Paulo, Ribeirão Preto, SP, Brasil, Faculdade de Filosofia, Ciências e Letras de Ribeirão Preto, Universidade de São Paulo, Ribeirão Preto, SP, Brazil.

**Keywords:** *Streptococcus mutans*, oral pathogens, cariogenic bacteria

## Abstract

In Brazilian folk medicine, *Tetradenia riparia (*Hochst.) Codd. (Lamiaceae) is used to treat toothaches and dental abscesses and diseases induced by worms, bacteria, or fungi. This paper aims to investigate the chemical composition and the antibacterial effects of the essential oil obtained from *Tetradenia riparia* leaves (TR-EO) grown in Southeastern Brazil against a representative panel of oral pathogens. We evaluated the antibacterial activity of TR-EO in terms of the minimal inhibitory concentration (MIC). We identified aromadendrene oxide (14.0%), (*E,E*)-farnesol (13.6%), dronabinol (12.5%), and fenchone (6.2%) as the major constituents of TR-EO. TR-EO displayed MIC values between 31.2 and 500 μg/mL, with the lowest MIC value being obtained against *Streptococcus mitis* (31.2 μg/mL), *S. mutans* (62.5 μg/mL), *S. sobrinus* (31.2 μg/mL), and *Lactobacillus casei* (62.5 μg/mL). In time-kill experiments, TR-EO demonstrated bactericidal activity against *S. mutans* within the first 12 h, resulting in a curve profile similar to that of chlorhexidine. These results revealed that the essential oil of *Tetradenia riparia* displays promising activity against most of the selected cariogenic bacteria, including *Streptococcus mutans*.

## Introduction

Dental caries is a major public health concern that affects many countries worldwide. This pathology and other periodontal diseases are associated with acidogenic and aciduric bacteria that adhere to the tooth surface as a structurally and functionally organized biofilm (dental plaque) (Marsh, 2003; Marsh, 2006). The most efficient procedure to prevent caries is to remove the biofilm by brushing and flossing; however, most people fail to maintain a sufficient level of control through mechanical removal only (Barnett, 2006). Therefore, the use of oral products containing antimicrobial ingredients as a complementary measure has become necessary and has great value in regard to diminishing tooth surface biofilm (Furiga *et al.*, 2008; Sharma *et al.*, 2004). Currently, chlorhexidine is considered to be the anticariogenic gold standard and has received the approval of the American Dental Association Council on Dental Therapeutics. Nevertheless, the regular use of oral care products containing this chemical often incurs several side effects (Greenberg *et al.*, 2008; More *et al.*, 2008). As a result, the search for new potential chemotherapeutic agents that can be incorporated into dental products has escalated in recent years (Palombo, 2011).

Over the last decade, a number of papers have reported the antimicrobial potential of essential oils (EOs) extracted from plants against oral pathogens (Aguiar *et al.*, 2013; Alviano *et al.*, 2005; Botelho *et al.*, 2007; Filoche *et al.*, 2005; Iscan *et al.*, 2002; Maggi *et al.*, 2009). EOs consist of mixtures of a variety of lipid-soluble and volatile compounds, such as monoterpenes, sesquiterpenes, and phenylpropanoids, that can easily diffuse across cell membranes, a major advantage with regard to interactions with intracellular targets (Edris, 2007). Additionally, synergistic interactions between the components of EOs are possible and beneficial for their activities (Dorman and Deans, 2000).

*Tetradenia riparia* (Hochst.) Codd. (Lamiaceae), commonly known in Brazil as "false myrrh", is an herbaceous and aromatic shrub that originated in South Africa; it was introduced as an exotic ornamental plant in Brazil (Gazim *et al.*, 2010; Phillipson and Steyn, 2008). In folk medicine, this species is used to treat toothaches and dental abscesses, malaria, and diseases induced by worms, bacteria, or fungi, among others (Scott *et al.*, 2004; Vanpuyvelde *et al.*, 1988; Vlietinck *et al.*, 1995). The essential oil from *T. riparia* leaves displays repellent (Omolo *et al.*, 2004), insecticidal (Dunkel *et al.*, 1990), ascaricidal (Peter and Deogracious, 2006), antimalarial (Campbell *et al.*, 1997), and antinociceptive actions (Gazim *et al.*, 2010). Recently, the antimicrobial activity of this oil against *Candida albicans*, *Staphylococcus aureus*, *Bacillus subtilis*, *Escherichia coli*, *Pseudomonas aeruginosa*, *Enterococcus faecalis*, *Proteus mirabilis*, *Klebsiella pneumonia*, and *Salmonella enterica* was reported (Gazim *et al.*, 2010). However, despite its use in folk medicine to treat toothaches and dental abscesses, the effects of this essential oil against oral pathogens have not yet been investigated.

This paper reports the chemical composition and antimicrobial activity of the essential oil of *T. riparia* leaves (TR-EO) grown in Southeastern Brazil against a representative panel of cariogenic bacteria.

## Materials and Methods

### Plant material

*Tetradenia riparia* (Hochst.) Codd. (Lamiaceae) was collected at "Sítio 13 de Maio" (20°26′ S 47°27′ W, 977 m) in February 2010 near Franca, State of São Paulo, Brazil and identified by Prof. Milton Groppo. A voucher specimen (SPFR12421) was deposited at the Herbarium of Departamento de Biologia, Faculdade de Filosofia, Ciências e Letras de Ribeirão Preto, Universidade de São Paulo, São Paulo, Brazil (Herbarium SPFR).

### Essential oil extraction, GC and GC-MS analysis

Fresh leaves (300 g) were submitted to hydrodistillation in a Clevenger-type apparatus for 3 h. To this end, 1,200 g of plant material was divided into three samples of 400 g each, and 500 mL of distilled water was added to each sample. After manual collection, traces of water remaining in the essential oil (EO) were removed using anhydrous sodium sulfate, which was followed by filtration. The EO was stored in an amber bottle and kept in the refrigerator at 4 °C until further analysis. The EO yield was calculated from the weight of fresh leaves and expressed as the average of triplicate analysis.

### GC-FID and GC-MS analyses

TR-EO was analyzed by gas chromatography (GC) on a Hewlett-Packard G1530A 6890 gas chromatograph fitted with FID and a data-handling processor. An HP-5 (Hewlett-Packard, Palo Alto, CA, USA) fused-silica capillary column (30 m × 0.25 mm i.d.; 0.33 μm film thickness) was employed. The operation conditions were as follows: column temperature programmed to rise from 60 to 240 °C at 3 °C/min and then held at 240 °C for 5 min; carrier gas = H_2_, at 1.0 mL/min; injection mode; injection volume, 0.1 μL (split ratio of 1:10); and injector and detector temperatures = 240 and 280 °C, respectively. Components relative concentrations were obtained by peak area normalization (%). The relative areas were the average of triplicate GC-FID analyses.

GC-MS analyses were carried out on a Shimadzu QP2010 Plus (Shimadzu Corporation, Kyoto, Japan) system equipped with an AOC-20i autosampler. The column was a Rtx-5MS (Restek Co., Bellefonte, PA, USA) fused silica capillary column (30 m × 0.25 mm i.d. × 0.25 μm film thickness). Electron ionization mode occurred at 70 eV. Helium (99.999%) was employed as the carrier gas at a constant flow of 1.0 mL/min. The injection volume was 0.1 μL (split ratio of 1:10). The temperatures of the injector and the ion-source temperature were set at 240 and 280 °C, respectively. The oven temperature program was the same as the program used for GC. Mass spectra were taken with a scan interval of 0.5 s, in the mass range from 40 to 600 Da. TR-EO components identification was based on their retention indices on an Rtx-5MS capillary column under the same operating conditions as in the case of GC relative to a homologous series of *n*-alkanes (C_8_-C_24_); structures were computer-matched with the Wiley 7, NIST 08, and FFNSC 1.2 spectra libraries, and their fragmentation patterns were compared with literature data (Adams, 2007). Standard compounds available in our laboratory were also co-eluted with TR-EO to confirm the identity of some essential oil components.

### Bacterial strains and antimicrobial assays

The TR-EO minimum inhibitory concentration (MIC) values were calculated by using the broth microdilution method in 96-well microplates (CLSI, 2009). The following standard strains from the ATCC were used: *Streptococcus salivarius* (ATCC 25975), *Streptococcus sobrinus* (ATCC 33478), *Streptococcus mutans* (ATCC 25175), *Streptococcus mitis* (ATCC 49456), *Streptococcus sanguinis* (ATCC 10556), and *Lactobacillus casei* (ATCC 11578). Individual 24-hour colonies from blood agar (Difco Labs, Detroit, Mich, USA) were suspended in 10.0 mL of tryptic soy broth (Difco). Standardization of each microorganism suspension was carried out using a spectrophotometer (Femto, São Paulo, Brazil) at a wavelength (λ) of 625 nm, to match the transmittance of 81, equivalent to 0.5 on the McFarland scale (1.5 × 10^8^ cfu/mL), followed by dilution to a final concentration of 5 × 10^5^ cfu/mL. The samples were dissolved in DMSO (Merck, Darmstadt, Germany) at 4 mg/mL and were then diluted in tryptic soy broth (Difco), to yield concentrations between 3.9 and 4000 μg/mL. The final DMSO concentration was 5% (v/v), and this solution was used as a negative control. One inoculated well was included, to control broth adequacy for organism growth. One non-inoculated well free of antimicrobial agents was also included, to ensure medium sterility. Two-fold serial dilutions of chlorhexidine dihydrochloride (CHD) (Sigma-Aldrich, St. Louis) were made in tryptic soy broth (Difco), to obtain concentrations ranging from 59.0 to 0.115 g/mL. These dilutions were used as positive controls. The microplates (96 well) were sealed with parafilm and incubated at 37 °C for 24 h. Before the addition of resazurin and the determination of the minimal bactericidal concentration (MBC), an aliquot of the inoculum was aseptically removed from each well presenting no apparent growth and then plated onto tryptic soy agar supplemented with 5% sheep blood. The plates were incubated as described above. After plating, 30 μL of 0.02% resazurinin aqueous solution (Sigma, St. Louis, MO, USA) was poured into each microplate reservoir, to indicate microorganism viability (Porto *et al.*, 2009). The minimal inhibitory concentration (MIC) was determined as the lowest EO concentration capable of inhibiting microorganism growth. Three replicates were made for each microorganism.

The determination of MBC values (the lowest EO concentration in which 99.99% or more of the initial inoculum was killed) and the TR-EO time-kill-assays were conducted against *S. mutans* only because it is considered one of the primary causative agents of dental caries (Chung *et al.*, 2006). Time-kill assays were performed in triplicate on the basis of the methodology established by D'Arrigo and co-workers (D'Arrigo *et al.*, 2010). Tubes containing TR-EO at final concentrations of 62.5, 125, and 187.5 μg/mL (respectively one, two, and three times the TR-EO minimum bactericidal concentration for *S. mutans*) were inoculated with the tested microorganism, which resulted in an initial bacterial density of 5 × 10^5^ cfu/mL, and then incubated at 37 °C. Samples were removed, to determine viable strains at 0, 30 min, 6, 12, and 24 h after incubation, followed by dilution in sterile fresh medium when necessary. The diluted samples (50 μL) were spread onto tryptic soy agar plate supplemented with 5% sheep blood, incubated at 37 °C, and counted after 48 h. Time-kill curves were constructed by plotting log_10_ cfu/mL vs time. The assays were conducted in triplicate for each concentration and also for the positive (CHD, 0.92 μg/mL) and negative controls (suspension of *S. mutans* without added TR-EO).

## Results

**Figure 1:**
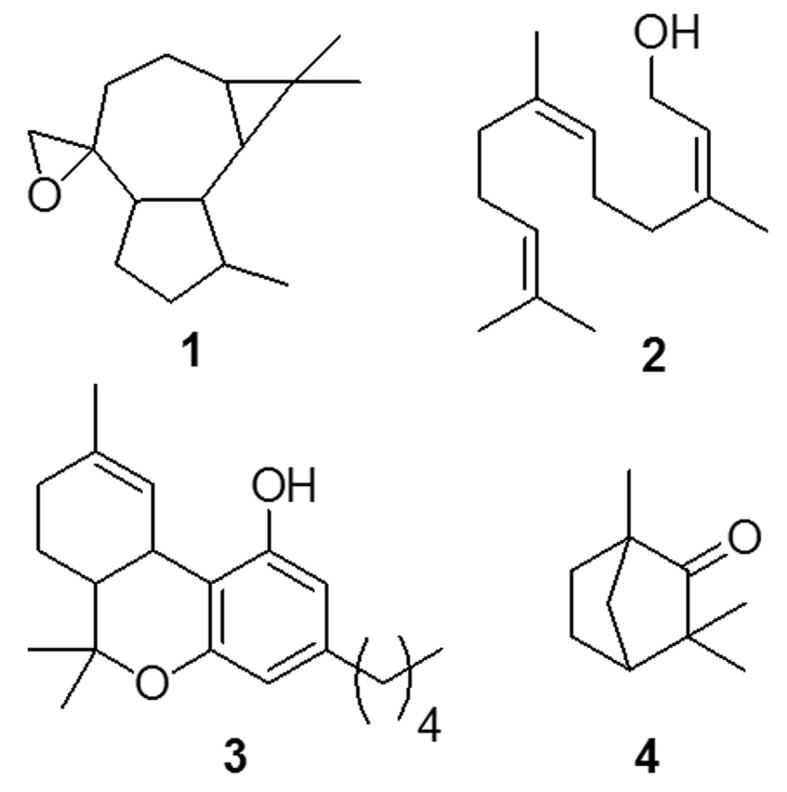
Chemical structures aromadendrene oxide (1), (*E,E*)-farnesol (2), dronabinol (3), and fenchone (4).

We obtained the essential oil extracted from *T. riparia* leaves (FV-EO) in 1.09 ± 0.15% yield (w/w). Table 1 depicts the chemical composition of TR-EO, as determined by GC-FID and GC-MS analyses. We identified a total of 37 compounds, with a predominance of oxygenated sesquiterpenes (42.7%). We verified that aromadendrene oxide (1, 14.0%), (*E,E*)-farnesol (2, 13.6%), dronabinol (3, 12.5%), and fenchone (4, 6.3%) were the major constituents in TR-EO (Figure 1).

**Table 1:** Chemical composition of the essential oil of *Tetradeniariparia* leaves (TR-EO).

Compound	RT	RI_exp_	RI_lit_	RA %	Identification
α-pinene	6.53	938	939	t	RL, MS, Co
Camphene	6.95	954	953	0.6	RL, MS
Sabinene	7.62	978	976	0.8	RL, MS
β-pinene	7.75	983	980	0.5	RL, MS, Co
Limonene	9.35	1033	1031	0.9	RL, MS, Co
*cis*-β-ocimene	9.60	1040	1043	0.5	RL, MS
Fenchone (4)	11.51	1095	1094	6.3	RL, MS, Co
α-fenchol	12.44	1120	1104	0.7	RL, MS
Camphor	13.64	1151	1143	2.0	RL, MS, Co
Borneol	14.46	1173	1165	0.8	RL, MS
Terpinen-4-ol	14.89	1184	1177	0.7	RL, MS
α-terpineol	15.41	1197	1189	1.0	RL, MS
Unknown	21.12	1372	-	0.4	
Unknown	22.63	1391	-	0.8	
α-copaene	23.25	1399	1376	0.8	RL, MS
β-elemene	23.94	1416	1391	1.5	RL, MS
α-gurjunene	24.33	1426	1409	3.8	RL, MS
*Trans*-caryophyllene	24.89	1441	1428	1.1	RL, MS, Co
α-*trans*-bergamotene	25.59	1460	1436	0.3	RL, MS
α-humulene	25.87	1467	1467	0.4	RL, MS, Co
Aromadendrene	26.73	1490	1491	0.6	RL, MS
Unknown	26.90	1495	-	0.6	
Viridiflorene	27.10	1500	1493	0.9	RL, MS
*E*, *E*-α-Farnesene	27.22	1503	1508	2.7	RL, MS
Bicyclogermacrene	27.33	1507	1517	0.4	RL, MS
α-muurolene	27.59	1510	1510	0.5	RL, MS
Unknown	27.79	1519	-	1.4	
4-Methyl-2 6-di-*tert*-butylphenol	27.91	1523	1519	0.4	RL, MS, Co
Cadinene	28.05	1527	1513	2.1	RL, MS
*Cis*-nerolidol	28.19	1531	1539	1.5	RL, MS
Unknown	28.27	1540	-	0.6	
Germacrene-D-4-ol	30.08	1582	1574	5.0	RL, MS
Spathulenol	30.16	1585	1576	0.1	RL, MS
Viridiflorol	30.36	1590	1590	2.0	RL, MS
Unknown	31.04	1610	-	0.8	
α-cadinol	32.41	1650	1653	2.6	RL, MS
α-Muurolol	32.56	1655	1657	0.3	RL, MS
Unknown	32.71	1659	-	1.5	
t-cadinol	32.89	1664	1660	5.1	RL, MS
Aromadendrene oxide (1)	33.35	1672	1668	14.0	RL, MS
*E*, *E*-Farnesol (2)	34.18	1702	1706	13.6	RL, MS
13-epimanoyl oxide	43 29	1996	2002	5.9	RL, MS
Cembrene C	43.39	2000	2005	0.2	RL, MS
Unknown	47.45	2138	-	1.2	
Dronabinol (3)	48.88	2190	2202	12.5	RL, MS
Unknown	53.18	2353	-	1.0	
Total				99.9	
Monoterpene hydrocarbons	3.3				
Oxygenated monoterpenes	11.5				
Sesquiterpene hydrocarbons	15.1				
Oxygenated sesquiterpenes	42.7				
Others	19.0				
Not identified	8.3				

RI_exp_: Retention index determined relative to *n*-alkanes (C_8_-C_20_) on the Rtx-5MS column. b) RI_lit_: Retention index from the literature (Adams, 2007). c) Calculated from the peak area relative to the total peak area. d) Compound identification: RL, comparison of the RI with those of the literature (Adams, 2007); RA: relative area (peak area relative to the total peak area in the GC-FID chromatogram), average of three replicates; MS, comparison of the mass spectra with those of the Wiley 7, NIST 08, and FFNSC 1.2 spectral libraries as well as with those of literature (Adams, 2007); Co: co-elution with standard compounds available in our laboratory; t: relative area lower than 0.1%.

We investigated the antibacterial activity of TR-EO against the main cariogenic bacteria in terms of their minimum inhibitory concentrations (MIC) values compared with chlorhexidine dihydrochloride (CHD, positive control). Table 2 summarizes the obtained MIC values. TR-EO furnished MIC values ranging from 31.2 to 500 μg/mL against the main causative agents of dental caries. The lowest TR-EO MIC values were obtained against *S. mitis* (31.2 μg/mL), *S. mutans* (62.5 μg/mL), *L. casei* (62.5 μg/mL), and *S. sobrinus* (62.5 μg/mL).

**Table 2:** Minimum inhibitory concentration (MIC) values (μg/mL) of the essential oil of *Tetradeniariparia* (TR-EO) against selected cariogenic bacteria.

Tested bacteria	TR-EO	CHD
*Streptococcus mutans*	62.50	0.92
*Streptococcus mitis*	31.25	3.68
*Lactobacillus casei*	62.50	0.92
*Streptococcus sanguinis*	125.0	7.37
*Streptococcus sobrinus*	62.50	0.92
*Streptococcus salivarus*	125.0	0.92

CHD: chlorhexidine dihydrochloride.

**Figure 2:**
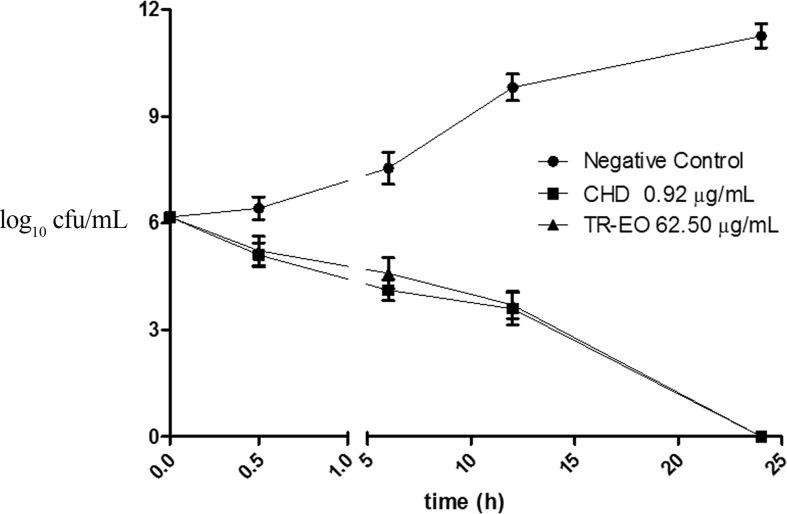
Time-kill curve for the essential oil of *T. riparia* (TR-EO) against *S. mutans* (5 × 10^5^ cfu/mL). CHD: chlorhexidine.

Analysis of Figure 2 revealed that (1) at its MBC (62.5 μg/mL), TR-EO exhibited a bactericidal effect against *S. mutans*, the main cariogenic bacteria, within the first 12 h and that (2) its action became more pronounced after this period. We also constructed time-kill curves using two and three times the MBC value (data not shown). However, we did not verify any significant differences between the periods, indicating that no dose-dependent response effects existed for TR-EO in the assays conditions (p < 0.05). Moreover, the TR-EO and CHD time-kill curve profiles were very similar.

## Discussion

Campbell and co-workers have previously investigated the *in vitro* antimalarial activity and the chemical composition of the essential oil from *Tetradenia riparia* leaves collected in South Africa (Campbell *et al.*, 1997), and they identified the monoterpenes α-terpineol (22.6%), fenchone (13.6%), β-fenchyl alcohol (10.7%), and perilla alcohol (6.0%) as major constituents. However, Omolo and co-workers identified fenchone (64.8%) and limonene (2.0%) as the main constituents of the repellent essential oil of *T. riparia* collected in Kenya (Omolo *et al.*, 2004). More recently, Gazim and co-workers investigated the seasonal variation in the chemical composition and the antimicrobial activity of the essential oil from *T. riparia* leaves collected in Southern Brazil (Gazim *et al.*, 2010) and verified that the most prevalent compounds in different seasons were the monoterpene fenchone; the sesquiterpenes 14-hydroxy-9-*epi*-caryophyllene, *cis*-muurolol-5-en-4-α-ol, and α-cadinol; and the diterpene calyculone. In the present study, we also detected fenchone and aromadendrene oxide in the essential oil of *T. riparia* leaves (Gazim *et al.*, 2010), but this is the first time that the presence of (*E,E*)-farnesol and dronabinol in this essential oil (Table 1) has been reported.

According to Rios and Recio (Rios and Recio, 2005) and Gibbons (Gibbons, 2004), EOs with MIC values higher than 1 mg/mL can be considered poorly active. However, EOs with MIC values below 100 μg/mL are interesting and very promising in the search for new antimicrobial agents. On the basis of these criteria and the data presented in Table 2, TR-EO MIC values ranged from 31.2 to 500 μg/mL against the main causative agents of dental caries. Among all of the tested bacteria, TR-EO gave one of the lowest MIC values against *S. mutans* (62.5 μg/mL). This is a noteworthy result because very few natural compounds are known to inhibit this microorganism, which is one of the primary causative agents of dental caries (Porto *et al.*, 2009; Saleem *et al.*, 2010).

The very promising MIC value of TR-EO against the main bacterial strain that causes caries disease (*S. mutans*) prompted us to investigate further aspects of the antimicrobial activity of this natural product, such as its minimal bactericidal concentration (MBC) and time-kill curve (Figure 2). Analysis of Figure 1 revealed that at its MBC, TR-EO exhibited its bactericidal effect within the first 12 h, and its action became more pronounced after this period. It is noteworthy that the time-kill curve profiles of TR-EO and CHD were very similar.

In the literature, two possible action mechanisms have been proposed to explain the biological activities of essential oils. Both mechanisms are associated with the hydrophobicity of monoterpenes and sesquiterpenes, which often are the main chemicals thereof. The hydrophobicity of terpenoids would allow these compounds to permeate the cell membranes easily, hence causing parasites or microorganisms death by affecting their metabolic pathways or organelles (Knobloch *et al.*, 1989). These essential oils themselves could interact with the parasite membrane and cause drastic physiological changes, leading to reduced membrane permeability and culminating in cell death (Bakkali *et al.*, 2008; Knobloch *et al.*, 1989). However, considering the large number of chemical constituents and synergistic or antagonistic interactions between these constituents, the essential oils could also act on cellular targets other than cell membranes, such as lipids and proteins (Bakkali *et al.*, 2008; Borges *et al.*, 2012). In this context, the antimicrobial activity of TR-EO against the selected oral pathogens might be related to the sesquiterpene (*E,E*)-farnesol, one of the major constituents of the essential oil. This compound has been reported to be active *in vitro* against *S. sobrinus* and *S. mutans* at concentrations of 14 μg/mL and 20 μg/mL, respectively (Koo *et al.*, 2002). The *in vivo* antimicrobial activity of (*E,E*)-farnesol in rodent teeth has also been evaluated by Koo and co-workers (Koo *et al.*, 2002). The authors concluded that the topical application of this compound at a concentration of 1 mM caused a decrease in biomass accumulation and prevented *S. mutans* adherence, thus confirming the potential of (*E,E*)-farnesol in caries prevention. However, the mechanism by which TR-EO displayed antimicrobial activity and the compounds responsible for the essential oil activity are not clear if we consider only the data obtained in this study.

## Conclusion

In summary, the essential oil of *T. riparia* (TR-EO) displays promising antimicrobial activity against some cariogenic bacteria, including *Streptococcus mutans*, which is one of the main causative agents of dental caries. The TR-EO chemical composition is slightly different from that reported in previous studies. Taken together, our results suggest that this essential oil might be promising for the development of new oral care products. Further studies aiming to identify the active chemical constituents of TR-EO are underway.

## References

[B01] Adams R (2007). Identification of Essential Oils Components by Gas Chromatography/Mass
Spectrometry.

[B02] Aguiar GP, Carvalho CE, Dias HJ (2013). Antimicrobial activity of selected essential oils against
cariogenic bacteria. Nat Prod Res.

[B03] Alviano WS, Mendonça-Filho RR, Alviano DS (2005). Antimicrobial activity of *Croton cajucara* Benth
linalool-rich essential oil on artificial biofilms and planktonic
microorganisms. Oral Microb Immun.

[B04] Bakkali F, Averbeck F, Averbeck D (2008). Biological effects of essential oils - A review. Food Chem Toxicol.

[B05] Barnett ML (2006). The rationale for the daily use of an antimicrobial
mouthrinse. J Am Dent Assoc.

[B06] Borges AR, Aires JR, Higino TM (2012). Trypanocidal and cytotoxic activities of essential oils from
medicinal plants of Northeast of Brazil. Exp Parasitol.

[B07] Botelho MA, Nogueira NA, Bastos GM (2007). Antimicrobial activity of the essential oil from *Lippia
sidoides*, carvacrol and thymol against oral
pathogens. Braz J Med Biol Res.

[B08] Campbell WE, Gammon DW, Smith P (1997). Composition and antimalarial activity *in vitro*
of the essential oil of *Tetradenia riparia*. Planta Med.

[B09] Chung JY, Choo JH, Lee MH (2006). Anticariogenic activity of macelignan isolated from
*Myristica fragrans* (nutmeg) against
*Streptococcus mutans*. Phytomedicine.

[B10] CLSI (2009). Susceptibility testing of aerobic bacteria Approved
standard. CLSI document M7-A8.

[B11] D'Arrigo M, Ginestra G, Mandalari G (2010). Synergism and postantibiotic effect of tobramycin and
*Melaleuca alternifolia* (tea tree) oil against
*Staphylococcus aureus* and *Escherichia
coli*. Phytomedicine.

[B12] Dorman HJD, Deans SG (2000). Antimicrobial agents from plants: antibacterial activity of plant
volatile oils. J Appl Microb.

[B13] Dunkel F, Weaver D, VanPuyvelde L (1990). Population suppression effects of Rwandan medicinal plant,
*Tetradenia riparia* (Hochst.) Codd (Lamiaceae) on stored
grain and bean insects.

[B14] Edris AE (2007). Pharmaceutical and therapeutic potentials of essential oils and
their individual volatile constituents: a review. Phytother Res.

[B15] Filoche SK, Soma K, Sissons CH (2005). Antimicrobial effects of essential oils in combination with
chlorhexidine digluconate. Oral Microb Immun.

[B16] Furiga A, Lonvaud-Funel A, Dorignac G (2008). *In vitro* anti-bacterial and anti-adherence
effects of natural polyphenolic compounds on oral bacteria. J Appl Microb.

[B17] Gazim ZC, Amorim ACL, Hovell AMC (2010). Seasonal variation, chemical composition, and analgesic and
antimicrobial activities of the essential oil from leaves of
*Tetradenia riparia* (Hochst.) Codd in Southern
Brazil. Molecules.

[B18] Gibbons S (2004). Anti-staphylococcal plant natural products. Nat Prod Rep.

[B19] Greenberg M, Dodds M, Tian M (2008). Naturally occurring phenolic antibacterial compounds show
effectiveness against oral bacteria by a quantitative structure-activity
relationship study. J Agr Food Chem.

[B20] Iscan G, Kirimer N, Kurkcuoglu M (2002). Antimicrobial screening of *Mentha piperita*
essential oils. J Agr Food Chem.

[B21] Knobloch K, Pauli A, Iberl B (1989). Antibacterial and antifungal properties of essential oil
components. J Essent Oil Res.

[B22] Koo H, Rosalen PL, Cury JA (2002). Effects of compounds found in propolis on *Streptococcus
mutans* growth and on glucosyltransferase
activity. Antimicrob Agents Ch.

[B23] Maggi F, Bramucci M, Cecchini C (2009). Composition and biological activity of essential oil of
*Achillea ligustica* All. (Asteraceae) naturalized in
central Italy: ideal candidate for anti-cariogenic
formulations. Fitoterapia.

[B24] Marsh PD (2003). Plaque as a biofilm: pharmacological principles of drug delivery
and action in the sub- and supragengival environment. Oral Dis.

[B25] Marsh PD (2006). Dental plaque as a biofilm and a microbial community -
implications for health and disease. BMC Oral Health.

[B26] More G, Tshikalange TE, Lall N (2008). Antimicrobial activity of medicinal plants against oral
microorganisms. J Ethnopharmacol.

[B27] Omolo MO, Okinyo D, Ndiege IO (2004). Repellency of essential oils of some Kenyan plants against
A*nopheles gambiae*. Phytochemistry.

[B28] Palombo EA (2011). Traditional medicinal plant extracts and natural products with
activity against oral bacteria: potential application in the prevention and
treatment of oral diseases. Evid Based Complement Altern Med.

[B29] Peter W, Deogracious O (2006). The *in vitro* ascaricidal activity of selected
indigenous medicinal plants used in ethnoveterinary practices in
Uganda. Afr J Tradit Complem.

[B30] Phillipson PB, Steyn CF (2008). *Tetradenia* (Lamiaceae) in Africa: new species
and new combinations. Adansonia.

[B31] Porto TS, Rangel R, Furtado N (2009). Pimarane-type diterpenes: antimicrobial activity against oral
pathogens. Molecules.

[B32] Rios JL, Recio MC (2005). Medicinal plants and antimicrobial activity. J Ethnopharmacol.

[B33] Saleem M, Nazir M, Ali MS (2010). Antimicrobial natural products: an update on future antibiotic
drug candidates. Nat Prod Rep.

[B34] Scott G, Springfield EP, Coldrey N (2004). A pharmacognostical study of 26 South African plant species used
as traditional medicines. Pharm Biol.

[B35] Sharma N, Charges CH, Lynch MC (2004). Adjunctive benefit of an essential oil-containing mouthrinse in
reducing plaque and gingivitis in patients who brush and floss
regularly. J Am Dent Assoc.

[B36] Vanpuyvelde L, Dekimpe N, Ayobangira FX (1988). Wheat rootlet growth-inhibition test of Rwandese medicinal
plants: active principles of *Tetradenia riparia* and
*Diplolophium africanum*. J Ethnopharmacol.

[B37] Vlietinck AJ, Van Hoof L, Tott J (1995). Screening of hundred Rwandese medicinal plants for antimicrobial
and antiviral properties. J Ethnopharmacol.

